# Variant 2 of KIAA0101, antagonizing its oncogenic variant 1, might be a potential therapeutic strategy in hepatocellular carcinoma

**DOI:** 10.18632/oncotarget.16702

**Published:** 2017-03-30

**Authors:** Lijuan Liu, Youyi Liu, Xiaobei Chen, Miao Wang, Yan Zhou, Ping Zhou, Wenxin Li, Fan Zhu

**Affiliations:** ^1^ Department of Medical Microbiology, School of Medicine, Wuhan University, Wuhan 430071, P.R. China; ^2^ College of Life Sciences, Wuhan University, Wuhan 430072, P.R. China; ^3^ Department of Infectious Diseases, Ren-Min Hospital of Wuhan University, Wuhan 430060, P.R. China; ^4^ Hubei Province Key Laboratory of Allergy and Immunology, Wuhan 430071, P.R. China

**Keywords:** hepatocellular carcinoma, alternative splicing, KIAA0101 transcript variant 2, isoform-selective competitor, therapeutic drug

## Abstract

Hepatocellular carcinoma (HCC) is one of the most lethal malignant tumors worldwide and effective therapies, including molecular therapy, remain elusive. Our previous work demonstrates that oncogenic KIAA0101 transcript variant (tv) 1 promotes HCC development and might be a HCC therapeutic target. However, the function of another KIAA0101 variant, KIAA0101 tv2, remains unknown. In this study, we reported that KIAA0101 tv2 was highly expressed in adjacent non-tumorous liver tissues (NTs) compared to HCC tissues. *In vivo* and *in vitro* results showed that KIAA0101 tv2 decreased cell survival, colony formation, tumor xenografts, migration, and invasion, as well as induced cell cycle arrest and apoptosis. Interestingly, it could inhibit the function of KIAA0101 tv1 by partially down-regulating KIAA0101 tv1, acting similar to KIAA0101 tv1 short hairpin RNA (shRNA). Further studies illustrated that KIAA0101 tv2 could increase the activity of p53 by competing with KIAA0101 tv1 for P53 binding. In conclusion, KIAA0101 tv2 exerts anti-tumor activity in HCC and acts as an endogenous competitor of tumor-associated KIAA0101 tv1. KIAA0101 tv2 has a potential to work as a therapeutic drug targeting the KIAA0101 tv1 in HCC.

## INTRODUCTION

Liver cancer is the second most lethal malignance worldwide, with more than half a million new cases diagnosed annually [1]. Hepatocellular carcinoma (HCC) represents 90% of the total liver cancer cases, resulting in approximately 500,000 deaths per year [2]. Most patients with HCC are diagnosed with advanced disease due to its aggressive properties. Therapy options for most advanced HCC cases are limited. The multi-kinase inhibitor sorafenib is the only systemic therapy for advanced HCC, suggesting that molecular therapy could be effective for HCC. However, sorafenib treatment only extends the median life expectancy of patients for 1 year [3, 4]. As a result, it is urgent to explore specific molecular targeted therapies for HCC.

KIAA0101, which regulates DNA replication, DNA repair, cell cycle progression, and cell proliferation [5–9], is associated with the development of several cancers [9–13] and predicts poor progression in different types of cancers [13–16]. Suppression of KIAA0101 is likely to be a strategy for cancer therapy [9, 12, 17]. According to the data from the National Center for Biotechnology Information (NCBI), KIAA0101 (Gene ID: 9768) has two major transcript variants (tv), designated KIAA0101 tv1 and tv2. Currently, all the studies regarding KIAA0101 focus on KIAA0101 tv1. Our previous study elucidated that KIAA0101 tv1 promoted cell survival by inhibiting p53 in HCC cells [12]. However, there is no report on KIAA0101 tv2 function.

Alternative splicing (AS) of pre-mRNAs is an essential process for gene expression regulation and contributes to proteome diversity in eukaryotes. It is also a process frequently deregulated during cancer progression [18, 19]. An increasing number of studies have reported that alternatively spliced isoforms may have related, distinct or even opposing functions [20, 21]. Hence, isoform-selective inhibitors are emerging as potential components for precision medical treatments [22, 23]. Furthermore, accumulating evidence shows that aberrant AS may regulate gene functions by generating endogenous inhibitor isoforms [24–27], which might be a new strategy for isoform-selective inhibitors. However, there has been no report exploring whether KIAA0101 tv2 could be an endogenous KIAA0101 tv1 inhibitor or competitor. As a result, studies on the function of KIAA0101 tv2 and its role in KIAA0101 tv1 regulation might provide a novel strategy for HCC molecular therapy.

In this study, we explored the expression pattern of KIAA0101 tv2 in HCCs and adjacent non-tumorous liver tissues (NTs). Moreover, the function and potential molecular mechanism of KIAA0101 tv2 on inhibition of HCC progression were revealed. In addition, the KIAA0101 tv2 regulation of KIAA0101 tv1 and its elementary molecular mechanism were also investigated. This study provides novel insights into the development of innovative strategies for HCC therapy.

## RESULTS

### KIAA0101 tv2 is highly expressed in NTs compared to HCCs

**Figure 1:**
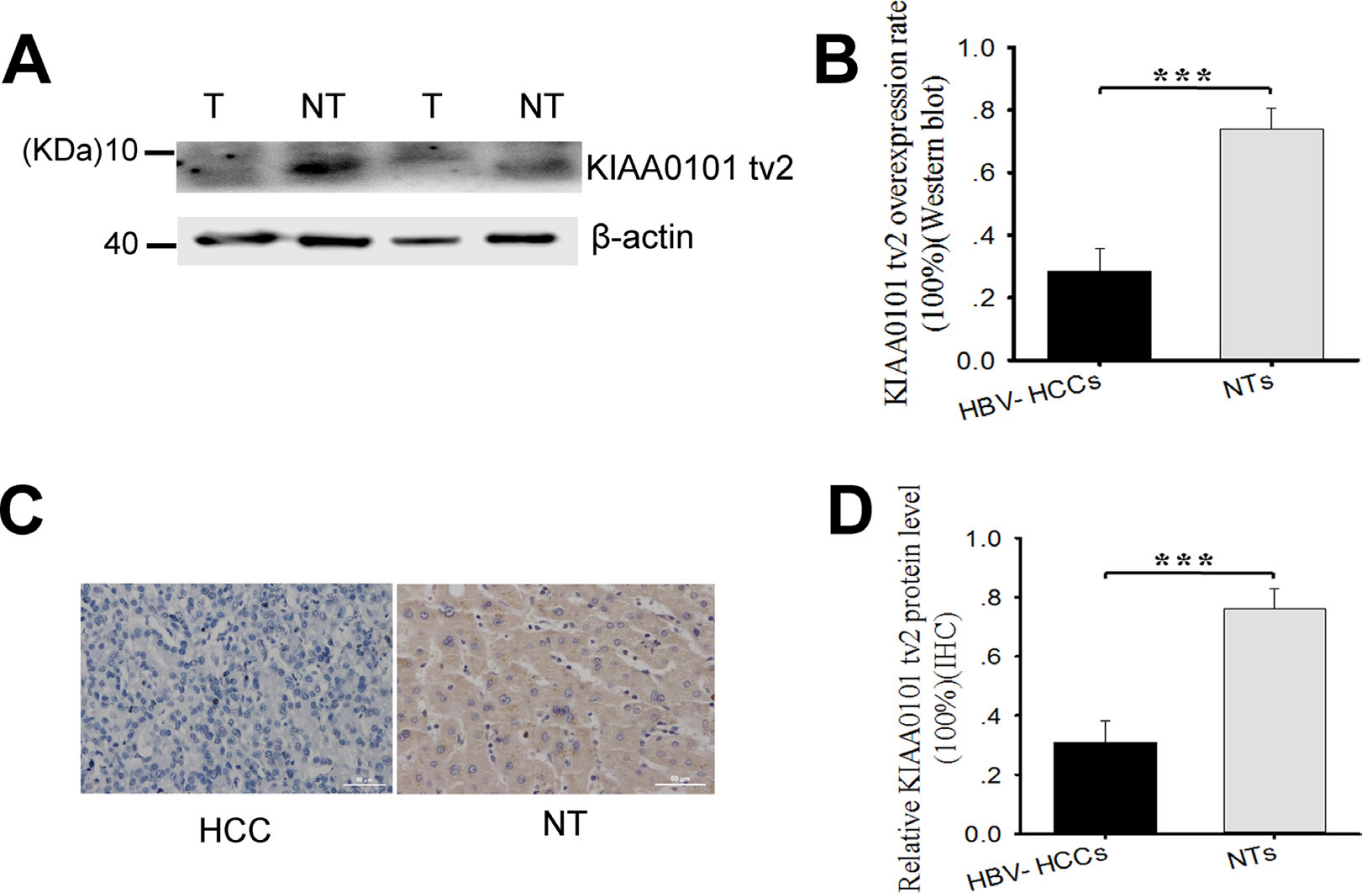
KIAA0101 tv2 is highly expressed in adjacent non-tumorous liver tissues (NTs) compared to hepatocellular carcinoma (HCC) tissues **(A-B)** KIAA0101 tv2 protein levels were analyzed by western blot in 42 pairs of HCC cases and NTs. The graph shows the mean±standard error of mean (SEM) of KIAA0101 tv2 protein overexpression rates by western blot. **(C-D)** All tissues were assessed by immunohistochemistry (IHC) using anti-KIAA0101 tv2 polyclonal antibody. Representative images are shown (Original magnification ×400). Each bar represents results from three independent experiments. ***P<0.005.

Western blot and immunohistochemistry (IHC) were performed to study the correlation between KIAA0101 tv2 expression and HCC using paired tissues from 42 patients. KIAA0101 tv2 was highly expressed in 73.8% (31/42) of NTs and only in 28.6% (12/42) of HCC tissues by western blot (Figure 1A-1B, Table 1, *P*<0.005). IHC also showed KIAA0101 tv2-positive expression in 76.2% (32/42) of NTs and in 31.0% (13/42) of HCC tissues (*P*<0.005) (Figure 1C-1D). These results indicate that KIAA0101 tv2 was highly expressed in NTs compared to HCC tissues.

**Table 1:** KIAA0101 tv2 protein levels were measured by western blot in 42 pairs of HCCs and NTs

Tissues Group	Number of Tissues	Number With KIAA0101 TV1 Overexpression	Ratio (%)	*P*
HCCs	42	31	73.8	<0.005
NTs	42	12	28.6	

### KIAA0101 tv2 shows opposite effect on cell proliferation and transformation compared with KIAA0101 tv1

**Figure 2:**
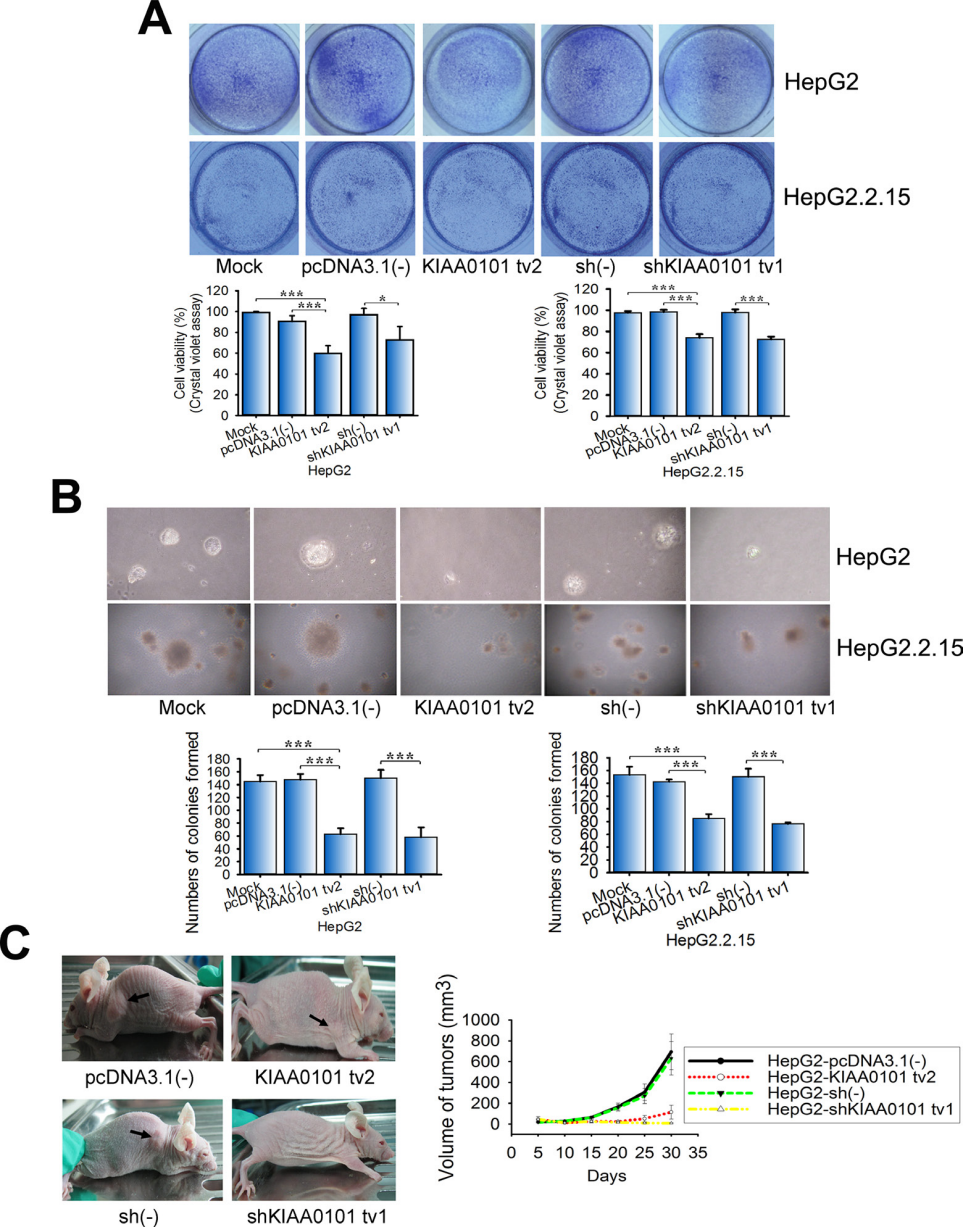
KIAA0101 tv2 inhibition of HCC cell proliferation and transformation is similar to short hairpin RNA (shRNA) targeted to KIAA0101 tv1 **(A)** The effect of KIAA0101 tv2 on HepG2 and HepG2.2.15 cell proliferation was examined by crystal violet assays. **(B)** Colony formation assay. KIAA0101 tv2 regulation of HepG2 and HepG2.2.15 cell transformation was demonstrated. **(C)** Tumor xenograft assay. Arrows indicate the xenograft tumors. The graph shows the mean±SEM of tumor volume induced by stable KIAA0101 tv2 or shKIAA0101 tv1-transfected HepG2 cells. All graphs show the mean±standard deviation (SD) **(A-B)** or mean±SEM **(C)** of at least three independent experiments. *P<0.05, ***P<0.005.

In our previous study, KIAA0101 tv1 was shown to be overexpressed in HCC tissues and exerted the properties of an oncogenic gene in HCC [12]. Because KIAA0101 tv2 was highly expressed in NTs compared to HCC, which was opposite to KIAA0101 tv1 expression, we explored whether the effects of KIAA0101 tv2 expression on cell proliferation and transformation were also opposite to those of KIAA0101 tv1. Crystal violet assays showed that KIAA0101 tv2 significantly inhibited the viability of NIH3T3 cells by 18.2% (P<0.001) compared to the control, whereas KIAA0101 tv1 increased the cell viability 1.5-fold (P<0.001) compared to the control (Supplementary Figure 1A). KIAA0101 tv2 function was also investigated in HCC cell lines (HepG2 and HepG2.2.15, which show abnormal expression of KIAA0101 tv1). Short hairpin RNA (shRNA) targeted to KIAA0101 tv1 (shKIAA0101 tv1) was used as a positive control. KIAA0101 tv2 significantly decreased the proliferation rates in HepG2 and HepG2.2.15 cells, at levels comparable to that in the control cells (Figure 2A). These results imply that KIAA0101 tv2 exhibits an opposing role to KIAA0101 tv1 on cell viability and proliferation.

Colony formation assays revealed that few foci were observed in NIH3T3-KIAA0101 tv2 cells compared to HepG2 cells (Supplementary Figure 1B, P<0.001). Similarly, fewer foci formed after transfection with KIAA0101 tv2 compared to empty vector in HepG2 and HepG2.2.15 cells using colony formation assays. A similar result was obtained in the positive control (Figure 2B, P<0.001). Furthermore, NIH3T3-KIAA0101 tv2 cells failed to form tumors on the flank of nude mice (Supplementary Figure 1C, P<0.001), indicating that KIAA0101 tv2 failed to induce NIH3T3 cell transformation. HepG2-KIAA0101 tv2 and HepG2-shKIAA0101 tv1 cells restricted tumor formation on the flank of nude mice (Figure 2C, Supplementary Table 1). These results implicate KIAA0101 tv2 similar to shKIAA0101 tv1 in the inhibition of HCC cell oncogenic potential.

### KIAA0101 tv2 is antagonistic to KIAA0101 tv1 during cell migration and invasion

**Figure 3:**
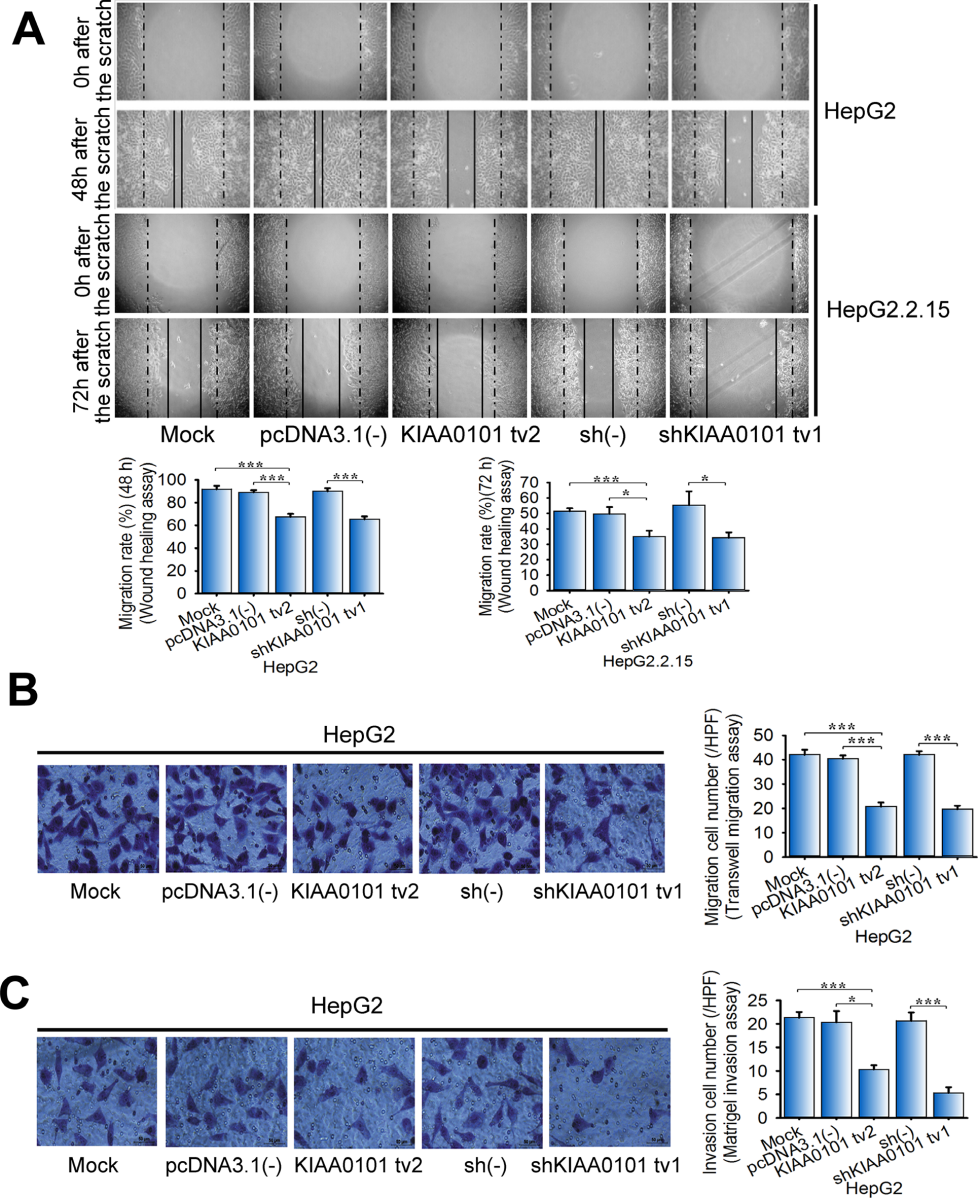
KIAA0101 tv2 suppresses HCC cell migration and invasion similar to shKIAA0101 tv1 **(A)** Wound healing assay. The effect of KIAA0101 tv2 on wound closure in HepG2 and HepG2.2.15 cells was determined. Representative wound closing cells (48 h/72 h) after onset of scratching (0 h) are shown. **(B)** Transwell migration assay and **(C)** Matrigel invasion assay were performed to determine the migration and invasion cell numbers respectively. Representative images of crystal violet-stained cells are shown (400×). The bars represent the mean±SD of three independent experiments performed in triplicate. *P<0.05, ***P<0.005.

There is increasing evidence that KIAA0101 promotes cell migration and invasion in several types of cancer cells, including renal carcinoma, gastric cancer, and esophageal cancer [10, 11, 15]. However, the roles of KIAA0101 tv2 in HCC cell migration and invasion are still unknown. Wound healing assays demonstrated that KIAA0101 tv2 overexpression inhibited wound closure in NIH3T3 cells by 35.1% (P=0.003<0.005). Conversely, KIAA0101 tv1 strongly improved the wound closure by 1.6-fold (P<0.001) (Supplementary Figure 2A). Overexpression of KIAA0101 tv2 significantly inhibited wound closure in HepG2 and HepG2.2.15 cells (P<0.05) (Figure 3A). This result was consistent with that of shRNA-mediated KIAA0101 tv1 knockdown, which showed significant reduction in migration rate (P<0.05) (Figure 3A).

Furthermore, both cell migration and invasion were enhanced by KIAA0101 tv1 overexpression in NIH3T3 cells (Supplementary Figure 2B-2C). In striking contrast, KIAA0101 tv2 overexpression significantly reduced cell migration and invasion in both NIH3T3 and HepG2 cells, as well as in KIAA0101 tv1-depleted HepG2 cells (Supplementary Figure 2B-2C and Figure 3B-3C).

Our results suggest that KIAA0101 tv2, unlike KIAA0101 tv1, fails to promote NIH3T3 cells migration and invasion. Moreover, KIAA0101 tv2 inhibits these malignant properties of HCC cells as shKIAA0101 tv1.

### Overexpression of KIAA0101 tv2 induces cell cycle arrest and apoptosis

**Figure 4:**
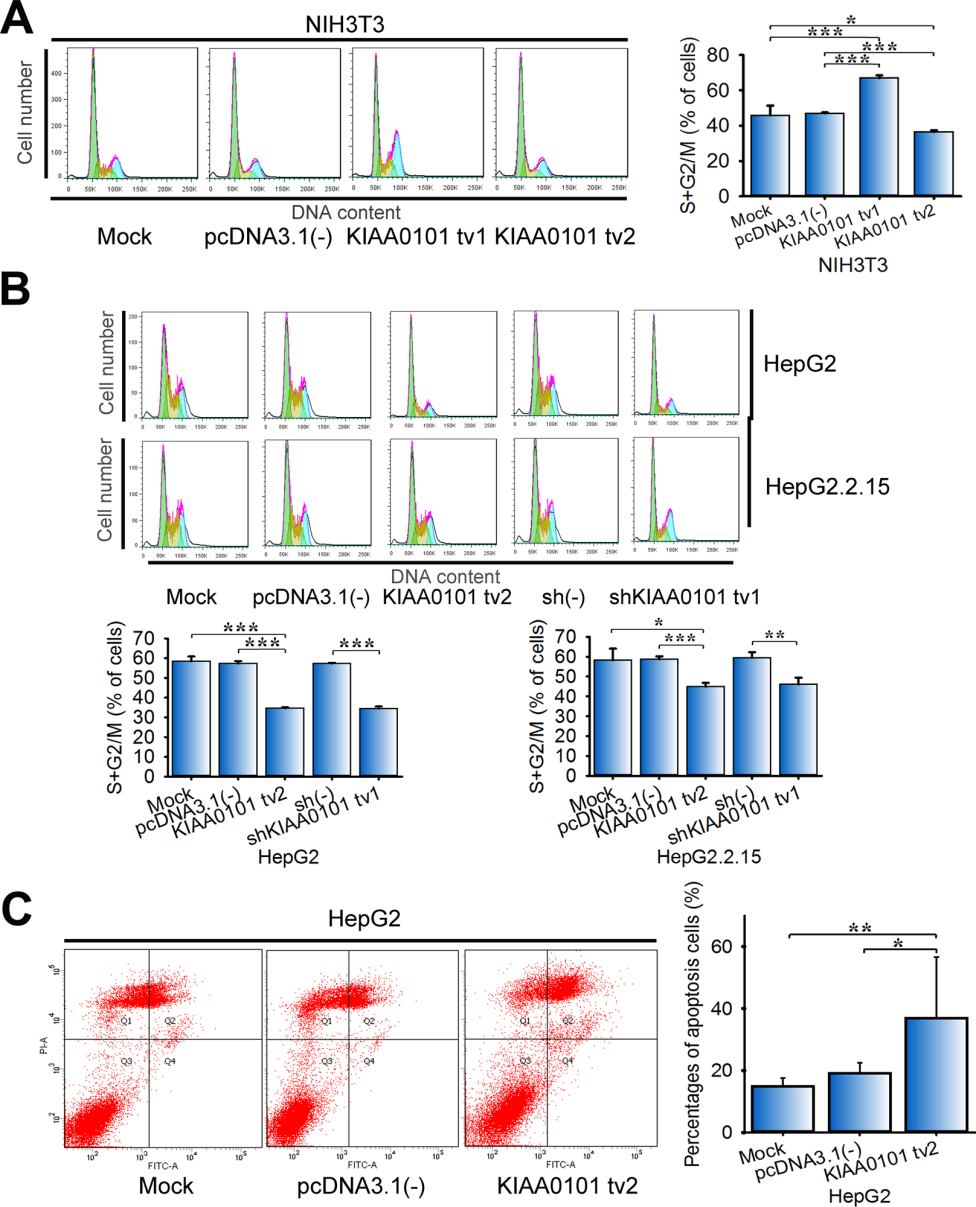
Overexpression of KIAA0101 tv2 induces cell cycle arrest and apoptosis **(A-B)** Cell cycle analysis. NIH3T3, HepG2, and HepG2.2.15 cells transfected with the indicated plasmids were harvested and fixed with ice-cold 70% ethanol at –20°C overnight. Cells were washed with PBS and incubated with propidium iodide (PI) containing RNase for 15 min. DNA content was determined by flow cytometry. *P<0.05, **P<0.01, or ***P<0.005 compared to the control group. **(C)** HepG2 cells stained with Annexin V/PI were used to detect apoptosis with a flow cytometer. All graphs show the mean±SD of at least three independent experiments. *P<0.05, **P<0.01.

Our previous study found that KIAA0101 tv1 protected HepG2 cells from doxorubicin-induced apoptosis [12]. Here we examined the role of KIAA0101 tv2 in the regulation of cell cycle and apoptosis. Flow cytometry revealed a 22.6% (P<0.001) decrease in the percentage of cells in the G2/M and S phases in NIH3T3-KIAA0101 tv2 cells. However, KIAA0101 tv1 overexpression dramatically increased the percentage of cells in the G2/M and S phases to 1.4-fold (P<0.001) (Figure 4A). Furthermore, KIAA0101 tv2 overexpression significantly decreased the percentage of HepG2 and HepG2.2.15 cells in G2/M and S phases (P<0.001), similar to shKIAA0101 tv1 in HepG2 and HepG2.2.15 cells (Figure 4B). Thus, KIAA0101 tv2, analogous to shKIAA0101 tv1, may plays a role in suppressing cell cycle progression.

Flow cytometry also showed a significant increase in the rate of apoptosis in KIAA0101 tv2-overexpressing HepG2 cells (Figure 4C, P<0.05). These results demonstrate that KIAA0101 tv2 overexpression can induce HCC cell cycle arrest and apoptosis.

### KIAA0101 tv2 overexpression down-regulates the expression of KIAA0101 tv1

**Figure 5:**
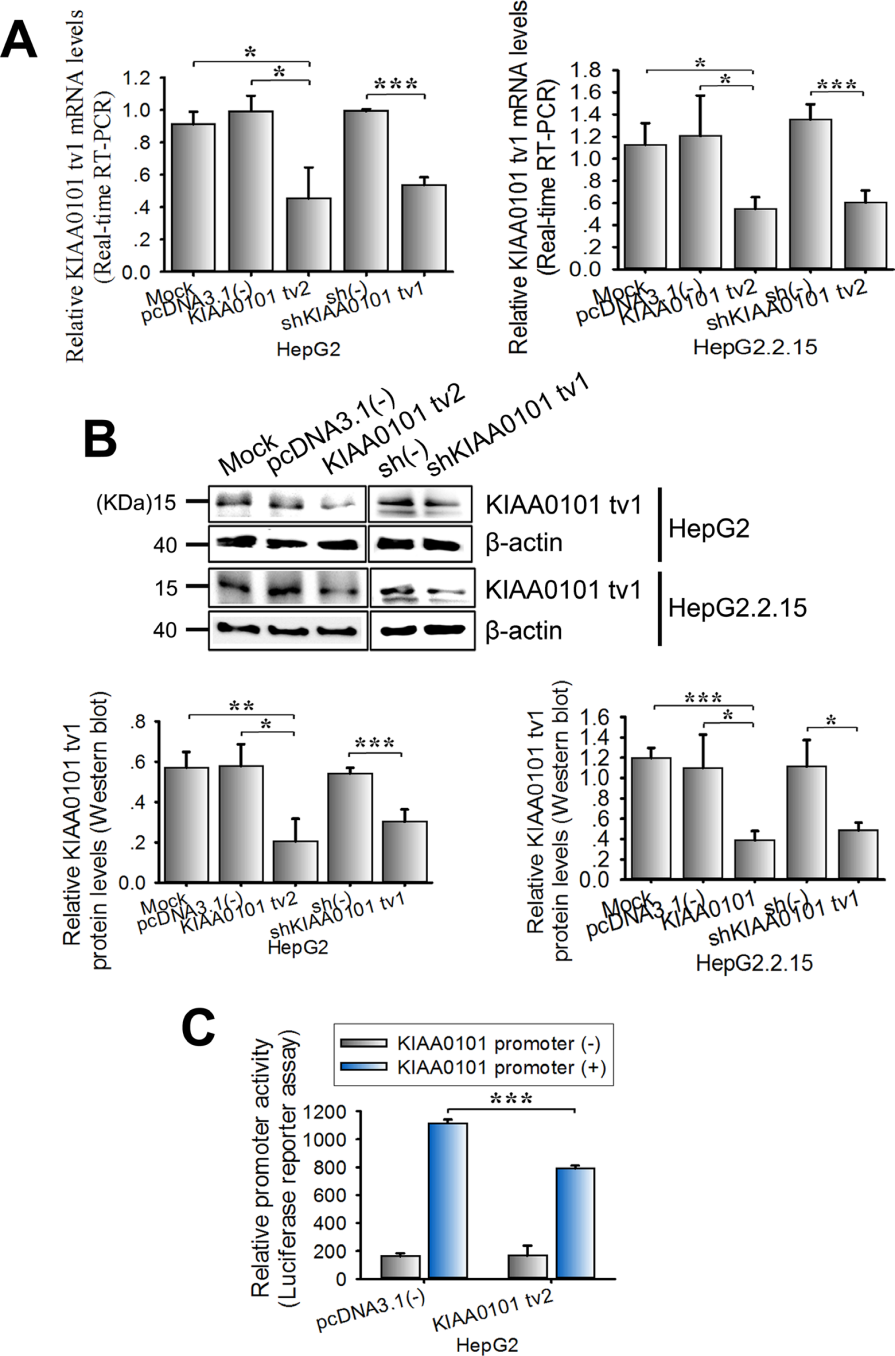
KIAA0101 tv2 overexpression suppresses the expression level of KIAA0101 tv1 in HCC cells HepG2 and HepG2.2.15 cells were transfected with KIAA0101 tv2 plasmid. shKIAA0101 tv1 was used as the positive control. pcDNA3.1(-) or scrambled shRNA (sh(-)) was used as the negative control. Mock transfection was used as the blank control. The mRNA and protein levels of KIAA0101 tv1 were determined in the indicated cells using **(A)** real-time RT-PCR and **(B)** western blot respectively. **(C)** Luciferase activity assay. KIAA0101 tv2 expression plasmids were transiently co-transfected with either pGL3-KIAA0101 promoter or pGL3-basic into HepG2 cells. Luciferase activity was measured following 48 h of incubation. All graphs show the mean±SD of at least three independent experiments. *P<0.05, **P<0.01, ***P<0.005.

Several studies report that AS could generate endogenous inhibitor isoforms [24–27]. Combined with our observations above, we explored whether the KIAA0101 tv1 expression levels were effected by KIAA0101 tv2 overexpression. As shown in Figure 5A-5B, overexpression of KIAA0101 tv2 significantly down-regulated the mRNA and protein levels of KIAA0101 tv1 in both HepG2 and HepG2.2.15 cells. These results suggest that KIAA0101 tv2 can down-regulate the expression of KIAA0101 tv1 in HCC cells.

To examine whether KIAA0101 tv2 effects the expression of KIAA0101 tv1 through regulating the KIAA0101 promoter activity, HepG2 cells were transiently co-transfected with the KIAA0101 promoter/luciferase reporter and pcDNA3.1(-)-KIAA0101 tv2 plasmid. Luciferase activity assays showed an approximately 34.6% (P<0.001) reduction in KIAA0101 promoter activity induced by KIAA0101 tv2 (Figure 5C). Therefore, KIAA0101 tv2 might inhibit the transcriptional activity of KIAA0101 in HepG2 cells in a negative feedback loop.

### KIAA0101 tv2 increases the transcriptional activity of p53 in HepG2 cells

**Figure 6:**
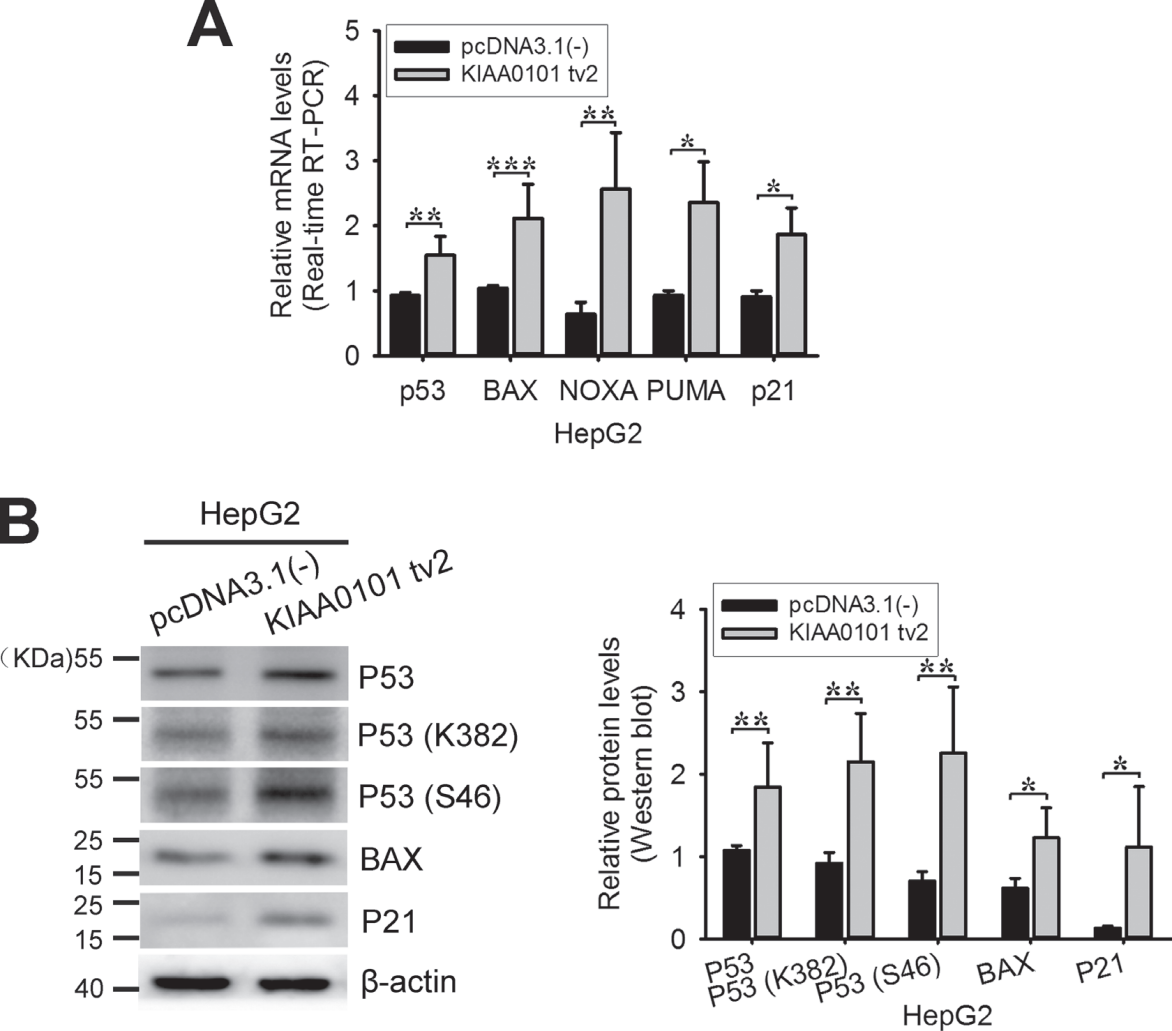
Exogenous expression of KIAA0101 tv2 increases p53 activity **(A)** HepG2 cells were transiently transfected with the empty plasmid or with the expression plasmid for KIAA0101 tv2. 48 hours after transfection, total mRNA was prepared and subjected to real-time RT-PCR using specific primers. β-actin was used as the control. The experiments were performed at least three times. **(B)** Western blot. The protein levels of P53, P53 (K382), P53 (S46), and two downstream target genes were assessed in pcDNA3.1(-) or KIAA0101 tv2 expression plasmid-transfected HepG2 cells. All bars show the intensity of the bands quantitated by densitometry based on three independent experiments. *P<0.05, **P<0.01, **P<0.005.

Our previous work showed that KIAA0101 tv1 suppressed the acetylation of p53 at Lys382 and inhibited the transcriptional activity of p53 [12]. In this study, we found KIAA0101 tv2 down-regulated the expression of KIAA0101 tv1 and promoted HepG2 cell apoptosis. To verify the relationship between these roles of KIAA0101 tv2 and the activity of cell signaling pathways, KIAA0101 tv2 was overexpressed in HepG2 cells. Then, we examined the changes in expression of the tumor suppressor p53 and its downstream target genes. As shown in Figure 6A-6B, the total expression levels of p53 and its target genes (such as BAX, NOXA, PUMA, and p21) were increased in HepG2-KIAA0101 tv2 cells. P53 modifications at both Ser46 and Lys382 are necessary for p53 transcription during apoptosis [28]. Furthermore, phosphorylation of P53 at S46 is required for P53-K382 acetylation [29]. Therefore, we also detected the level of P53-K382 acetylation and P53-S46 phosphorylation. Our results showed that KIAA0101 tv2 could increase both K382 acetylation and S46 phosphorylation of P53 (Figure 6B). These data indicate that KIAA0101 tv2 may exert an anti-tumor effect in HCC by increasing the transcriptional activity of p53.

### KIAA0101 tv2 competes with KIAA0101 tv1 for binding to p53 protein

**Figure 7:**
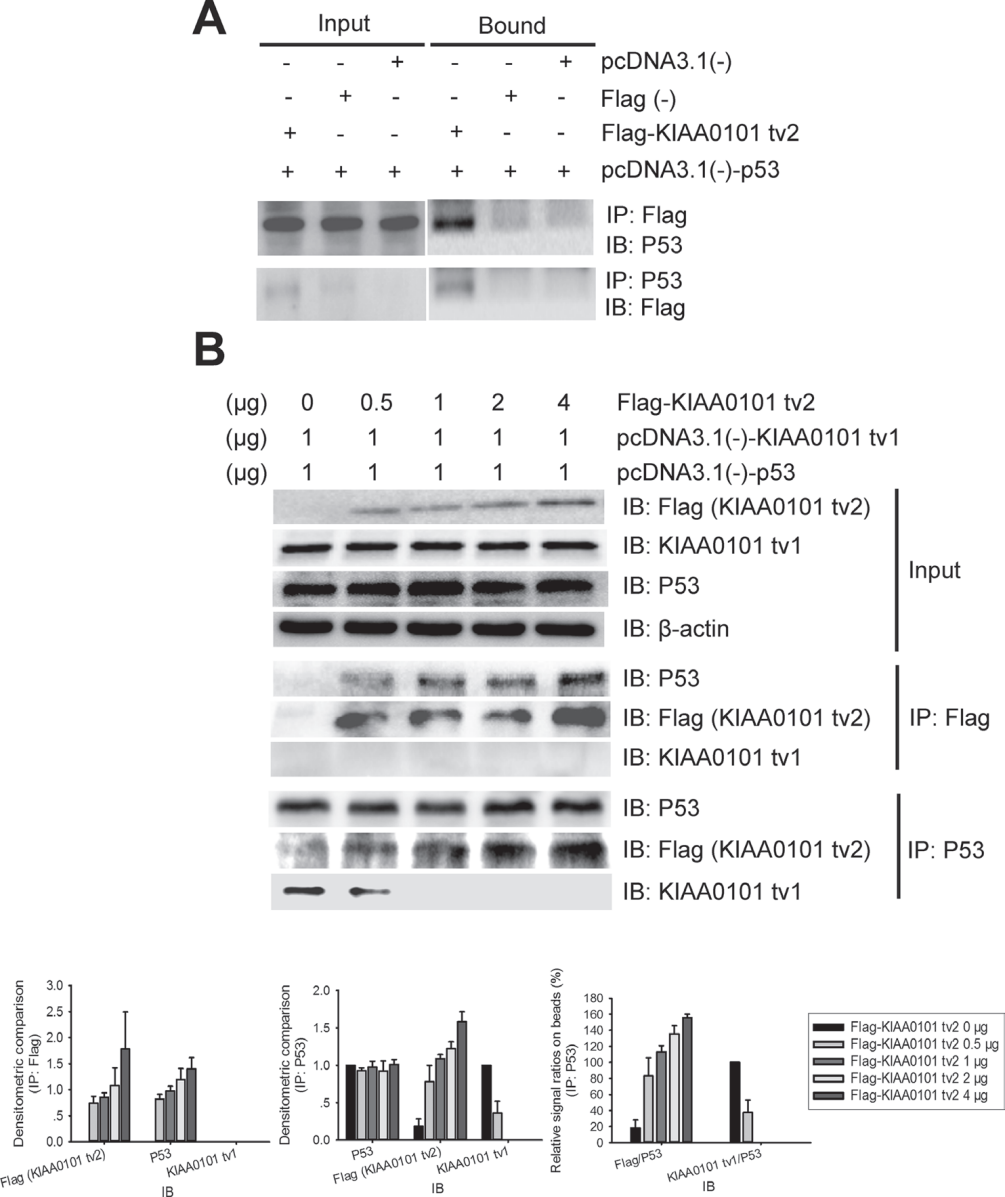
KIAA0101 tv2 competes with KIAA0101 tv1 for binding to P53 in mammalian cells **(A)** 48 hours after transfection, whole cell lysate was immunoprecipitated (IP) using anti-FLAG antibody and subsequently immunoblotted (IB) with anti-P53 antibody. The cell lysate was also immunoprecipitated using anti-P53 antibody and IB with the anti-FLAG antibody. IP lysate (5%) was used as input. **(B)** Increasing concentrations of KIAA0101 tv2 prevented the co-IP of KIAA0101 tv1 with P53. HEK 293T cells were co-transfected with pcDNA3.1(-)-KIAA0101 tv1 (1 μg), pcDNA3.1(-)-P53 (1 μg), and increasing amounts (0_4 μg) of Flag-KIAA0101 tv2 plasmids. Cell lysate was immunoprecipitated using the anti-FLAG antibody and subsequently IB with anti-P53 and anti-KIAA0101 tv1 antibodies. Cell lysate was also immunoprecipitated using anti-P53 antibody and then IB with antibodies against P53, Flag, and KIAA0101 tv1. Densitometric analysis presented as mean ± SEM. IP lysate (5%) was used as input.

Interactions between KIAA0101 tv2 and P53 were examined in HEK293T cells by immunoprecipitation analysis. As shown in Figure 7A, KIAA0101 tv2 and P53 interacted, suggesting that KIAA0101 tv2 forms a complex with P53.

In our previous study, KIAA0101 tv1 was shown to physically interact with the p53 protein [12]. Since both KIAA0101 tv2 and tv1 could bind to p53, we tested whether there was a competitive effect between KIAA0101 tv2 and tv1. The same amount of KIAA0101 tv1 and p53 plasmids were co-transfected with increasing amounts of Flag-tagged KIAA0101 tv2 plasmids into HEK293T cells. Increasing concentrations of Flag-KIAA0101 tv2 increased the interaction between P53 and KIAA0101 tv2 and decreased the binding between P53 and KIAA0101 tv1 (Figure 7B).

## DISCUSSION

KIAA0101, also called p15 (PAF), OEATC-1 (overexpressed in anaplastic thyroid carcinoma-1), or NS5ATP9 (HCV NS5A-transactivated protein 9), was initially identified as a proliferating cell nuclear antigen (PCNA) associated protein [30–32]. It is a transcription factor that regulates several target genes involved in the modulation of cellular processes such as DNA replication, DNA repair, proliferation and cell cycle regulation [5, 6, 9, 33]. KIAA0101 overexpression is associated with the development of several cancers, including pancreatic cancer [9], renal cell carcinoma [10], esophageal cancer [11], primary lung cancer [13], and HCC [16].

There are two main variants derived from the AS of KIAA0101 pre-mRNA (KIAA0101 tv1^+Ex3^; KIAA0101 tv2^-Ex3^) according to NCBI. Compared to KIAA0101 tv1, KIAA0101 tv2 lacks exon 3, which contains a conserved PCNA binding motif known as a PIP-box [32]. Transcripts from more than 90% of human multi-exon genes undergo AS [34]. Aberrant AS is proposed as a crucial mechanism in the development of cancer [20, 35]. Increasing evidence shows that spliced variants exert distinctive expression patterns in different types of cancers [36–38]. Our study also showed that KIAA0101 tv1 was overexpressed in HCCs compared to NTs, especially in late stage HCC [12], whereas KIAA0101 tv2 was highly expressed in NTs compared to HCCs (Figure 1 and Table 1), suggesting the expression pattern of KIAA0101 variants were opposite in HCC.

Differentially expressed variants in cancer and non-oncogenic cells may play disparate roles [38, 39], so we hypothesized that KIAA0101 tv2 might have distinct functions from tv1 in HCC. We previously found that KIAA0101 tv1 could promote NIH3T3 cells proliferation, colony formation, and tumor xenografts in nude mice [12]. It could also protect cells against doxorubicin-induced apoptosis [12]. Interestingly, our data showed KIAA0101 tv2 failed to promote NIH3T3 cells proliferation, colony formation, tumor xenografts in nude mice, cell motility, and invasion (Supplementary Figure 1, 2), suggesting KIAA0101 tv2 did not possess the oncogenic properties of KIAA0101 tv1. Additionally, several reports show the opposing roles of spliced variants from the same gene in cancer [26, 40, 41]. We also found KIAA0101 tv2 could induce cell cycle arrest and apoptosis, as well as inhibit the malignant properties in HCC cells (Figure 2, 3, 4), indicating that KIAA0101 tv2 might have tumor suppressor properties opposite to KIAA0101 tv1.

Alterations in the splicing patterns of genes may contribute to the regulation of gene functions by generating endogenous inhibitor isoforms [24–27]. For instance, Basigin-3 inhibits cell invasion by interacting with Basigin-2, which promotes cell invasion in HCC, as an endogenous inhibitor [26]. Interestingly, our data revealed that KIAA0101 tv2 inhibited the expression of KIAA0101 tv1 by suppressing the KIAA0101 promoter activity in HCC cells (Figure 5). The function of KIAA0101 tv2 is similar to shKIAA0101 tv1 in inhibiting the malignant potential of KIAA0101 tv1 in HCC (Figure 2, 3). Thus, our results imply that KIAA0101 tv2 might be the endogenous negative regulator of KIAA0101 tv1.

Endogenous regulators exert their function through regulation of the variants involved in signaling pathways [24, 25]. Our previous study showed that KIAA0101 tv1 inhibited the transactivation of p53 [12]. Hence, we explored whether KIAA0101 tv2 could regulate the transactivation of p53. In this study, we found that KIAA0101 tv2 overexpression significantly up-regulated the expression of total p53 and its target genes, the acetylation level of P53 at K382 and the phosphorylation level of P53-S46 (Figure 6). These results indicate that the p53 transactivation activity was increased, which mediated cell cycle arrest and apoptosis. The KIAA0101 tv2 regulation of p53 was opposite to tv1, as well [12]. KIAA0101 tv2 physically interacted with p53 by competing with KIAA0101 tv1 (Figure 7). KIAA0101 tv2 exerts anti-tumor activity in HCC as an endogenous competitor of oncogenic KIAA0101 tv1. KIAA0101 tv2 could be used as a negative regulator of KIAA0101 tv1 in HCC molecular therapy. In addition, it provides a potential isoform-selective therapeutic target for HCC treatment, which could improve efficacy while reducing the undesirable side effects.

In conclusion, KIAA0101 tv2 is overexpressed in NTs compared to HCC, and exerts different expression patterns from KIAA0101 tv1 in HCC tissues. It possesses tumor suppressor properties and inhibits the expression and function of KIAA0101 tv1 in HCC, similar to KIAA0101 tv1 shRNA. Consequently, KIAA0101 tv2 could serve as a competitor of KIAA0101 tv1 that suppresses the malignant properties of KIAA0101 tv1 in HCC development. Furthermore, developing novel isoform-selective drugs in HCC is a promising strategy, which will provide opportunities for precision medicine-based therapies. At the same time, it could also provide novel insights into developing innovative strategies for HCC therapy.

## MATERIALS AND METHODS

### Patients and tissue specimens

Paired HCC tissues from 42 patients were obtained from Ren-Min Hospital of Wuhan University in China from 2014 to 2016. Sample collections were under consensus agreements, and were approved by the Institutional Review Board of Wuhan University, School of Medicine. The samples were stored at –80°C until experiments were performed.

### Western blot

Protein samples (50 μg) were separated in 4% to 15% SDS-PAGE gel and transferred onto a nitrocellulose membrane (Amersharm Pharmacia Biotech, San Francisco, CA). Western blot was performed as formed standard procedures using the primary goat polyclonal antibody against KIAA0101 tv1 (ab126848, Abcam, Cambridge, UK), the primary rabbit polyclonal antibody against KIAA0101 tv2 (NBP-80555, Novus Biologicals, Littleton, CO), P53 (10442-1-AP, Proteintech, Chicago, IL, USA), P53 (acetyl K382) (ab75754, Abcam), P53 (phosphor S46) (ab76242, Abcam), BAX (50599-2-Ig, Proteintech), P21 (10355-1-AP, Proteintech). Peroxidase-conjugated anti-goat IgG (sc-2768, Santa Cruz, California, USA) and anti-rabbit IgG (7074, Cell Signaling Technology, MA, USA) were used as secondary antibody. Anti-β-actin-peroxidase monoclonal antibody (A3854, Sigma-Aldrich, Bedford, MA, USA) was used to probe the internal standard. For KIAA0101 tv2, the membranes were treated with 0.5% glutaraldehyde for 1 h before blocking and immunodetection. The immunoblot signal was visualized through enhanced chemiluminescence kit (Pierce Biotechnology, Rockford, IL, USA).

### Immunohistochemistry (IHC)

IHC stainings were carried out as described previously [12] using the primary rabbit polyclonal antibody against KIAA0101 tv2 (NBP-80555, Novus Biologicals). Peroxidase-conjugated anti-rabbit IgG (7074, Cell Signaling Technology) was used as secondary antibody.

### Plasmids construction

The KIAA0101 tv2 coding regions was cloned into the *BamH*I and *Hind*III sites of vector pcDNA3.1(-) (Invitrogen, Carlsbad, CA, USA) and pCMV-Tag 2B (Invitrogen) to generate pcDNA3.1(-)-KIAA0101 tv2 and Flag-KIAA0101 tv2 respectively using the primers P1-Forword and P1-Reverse (Supplementary Table 2).

The KIAA0101 promoter was amplified by primers P2-Forword and P2-Reverse, and then subcloned into the luciferase reporter vector pGL3-basic (Promega, Madison, WI, USA) to create pGL3-KIAA0101.

All constructs were confirmed by sequencing.

### Cell culture and transfection

HepG2 and mouse fibroblast cell line NIH3T3 were purchased from the Cell Bank of Shanghai institute of Cell Biology, Chinese Academy of Sciences (Shanghai, China) in 2011; HepG2.2.15 cell line (derived from HepG2 cells transfected with a plasmid carrying two head-to-tail copies of the HBV genome DNA serotype ayw, genotype D) was obtained from XiangYa Cell Center of Central South University (Changsha, China) in 2010. These cell lines were grown in Dulbecco's modified Eagle's medium (GIBCO BRL, MD, USA) containing 10% fetal bovine serum, 100 units/ml penicillin, and 100 μg/ml streptomycin sulfate at 37°C in 5% CO_2_; HepG2.2.15 cells were additionally maintained in medium containing 380 μg/ml G418 (Invitrogen; Carlsbad, CA, USA). These cell lines have been tested to be mycoplasm-free and authenticated by their examination of morphology and growth profile. HepG2 was additionally authenticated by the Cell Bank of Shanghai institute of Cell Biology, Chinese Academy of Sciences using short tandem repeat pro-filing.

Cells were transiently transfected with indicated plasmid(s) DNA for 48 h using Lipofectamine 2000 (Invitrogen) according to the manufacture's protocol.

### Crystal violet assay

Cells were lysed with 1% SDS for 30 min and absorbance was read at 570 nm using a spectrophotometer (Thermo Scientific Multiskan FC; Thermo Scientific, Wilmington, DE, USA). The percentage of cell survival was defined as the relative absorbance versus cells transiently transfected with empty vector.

### Colony formation assay and tumor xenograft assay

NIH3T3, HepG2 and/or HepG2.2.15 cells were transfected with indicated plasmid, and were maintained in 400 μg/ml of G418 at 24 h post transfection. G418-resistant colonies were isolated after 3-4 weeks of selection. The colony formation assay and tumor xenograft assay were performed as described previously [12].

### Wound healing assay

Cells were cultured to confluent in six-well plates. 24 h after transfection with indicated plasmids, cells were entertained serum starvation (DEME supplemented with 1% FBS) for another 24 h. Then linear wounds were created by scratching the monolayer with a standard 200 μl pipette tip. Cells migration into the wound area was monitored using an inverted phase contrast microscope (Olympus CH-40; Olympus, Tokyo, Japan) at the time the wound was created and each 24 h later. Four different equidistant points were measured for each plate. The migration rate was calculated as the proportion of the mean distance between both borderlines after closure versus the mean distance measured at 0 h for each plate.

### Transwell migration assay and matrigel invasion assay

Transwell migration assay and matrigel invasion assay were done using 24-well Transwell with an 8 μm diameter pore membrane (Costar, Cambridge, MA, USA) as described previously [42]. For matrigel invasion assay, Matrigel (BD Biosciences, San Jose, CA, USA) diluted to 200 μg/ml was used. After incubation at 37°C for 24 h, the cells adhered to the lower surface were fixed, stained with crystal violet, and counted under a light microscope (Olympus CH-40). Four fields in four separate quadrants of each membrane were counted and averaged.

### Flow cytometry

For cell cycle detection, approximately 10^6^ cells were harvested and fixed with ice-cold 70% ethanol at –20°C overnight. Cells were washed with PBS and incubated with staining solution containing 40 μg of propidium iodide (PI)/ml and 100 μg of RNase A/ml. After 15 min of incubation at 4°C, DNA content was determined by using a Beckman Coulter Epics Altra with Expo32 software (Beckman Coulter, Fullerton, CA, USA).

For apoptosis assay, the cells were stained with Annexin V/PI after washed with PBS. Then apoptosis was detected with a flow cytometer.

### RNA extraction and real-time reverse-transcriptase-PCR (RT-PCR)

Total RNA was extracted using TRIzol^®^ reagent (Invitrogen) according to the manufacturer's recommendation. RNA was treated with RQ1 RNase-Free DNase (Promega) and reverse transcribed using AMV-RT (Promega).

Quantification real-time RT-PCR was performed on the iCycler System (Bio-Rad, Hercules, CA, USA). Comparative quantification was used, normalizing KIAA0101 tv1, p53, BAX, NOXA, PUMA, and p21 to an internal standard gene (β-actin). The results were given as d-Ct values. The primers for β-actin were P3-Forword and P3-reverse (Supplementary Table 2). The primers for KIAA0101 tv1 were P4-Forword and P4-reverse. Specific primers for p53 were P5-Forword and P5-reverse. The primers for BAX were P6-Forword and P6-reverse. The primers for NOXA were P7-Forword and P7-reverse. The primers for PUMA were P8-Forword and P8-reverse, and the primers for p21 were P9-Forword and P9-reverse. The cycling conditions were 95°C for 2 minutes, then 95°C for 10 seconds, 58°C for 10 seconds, and 72°C for 10 seconds for 40 cycles.

### Luciferase reporter assay

The Dual-Luciferase^®^ reproter assay system (Promega) was used according to the manufacture's instruction. The activity of firefly luciferase was detected by a Luminometer. The transfection efficiency was normalized by *Renilla reniformis* activity.

### Immunoprecipitation

HEK293T cells were co-transfected with appropriate plasmids and incubated for 48 h. Cells were lysed in NTEP buffer (150 mM NaCl, 25 mM Tris-HCl, pH 7.5, 5 mM EDTA, 0.5% NP-40) plus freshly added 1× protease inhibitor cocktail (Sigma-Aldrich). Then Protein G Sepharose 4 Fast Flow beads (GE Heathcare, Piscataway, NJ, USA) were used to immunoprecipitate according to the manufacture's instruction. Immuno-complex precipitated by anti-FLAG antibody (66008-2-Ig, Proteintech) or anti-P53 antibody (10442-1-AP, Proteintech) were analysed by 4%-15% SDS-PAGE followed by western blot with anti-P53 or anti-FLAG or anti-KIAA0101 tv1 antibody.

### Statistical analysis

All experiments were performed in triplicate. SigmaPlot version 12.0 for Windows (Systat software Inc., USA) was used for the statistical analysis. All of these data were presented as mean±standard error of mean (SEM), or mean±standard deviation (SD). Statistical analysis was performed using χ^2^-test or Student's *t*-tests, with *P* < 0.05 taken as statistically significant. All the experiments were performed blindly.

## SUPPLEMENTARY MATERIALS FIGURES AND TABLES



## Abbreviations

HCC: hepatocellular carcinoma
tv: transcript variant
NTs: adjacent non-tumorous liver tissues
shRNA: short hairpin RNA
NCBI: National Center for Biotechnology Information
AS: alternative splicing
IHC: immunohistochemistry
OEATC-1: overexpressed in anaplastic thyroid carcinoma-1
NS5ATP9: HCV NS5A-transactivated protein 9
PCNA: proliferating cell nuclear antigen
RT-PCR: reverse-transcriptase-PCR
SEM: standard error of mean
SD: standard deviation
