# Supplementary material for: Variant 2 of KIAA0101, antagonizing its oncogenic variant 1, might be a potential therapeutic strategy in hepatocellular carcinoma
Source: Oncotarget. 2017 Mar 30;8(27):43990–4003. doi: 10.18632/oncotarget.16702 (PMC5546456; doi:10.18632/oncotarget.16702)
Supplement: Supplementary file 1 [file oncotarget-08-43990-s001.pdf]

Variant 2 of KIAA0101, antagonizing its oncogenic variant 1, might be a potential therapeutic strategy in hepatocellular carcinoma

SUPPLEMENTARY FIGURES AND TABLES

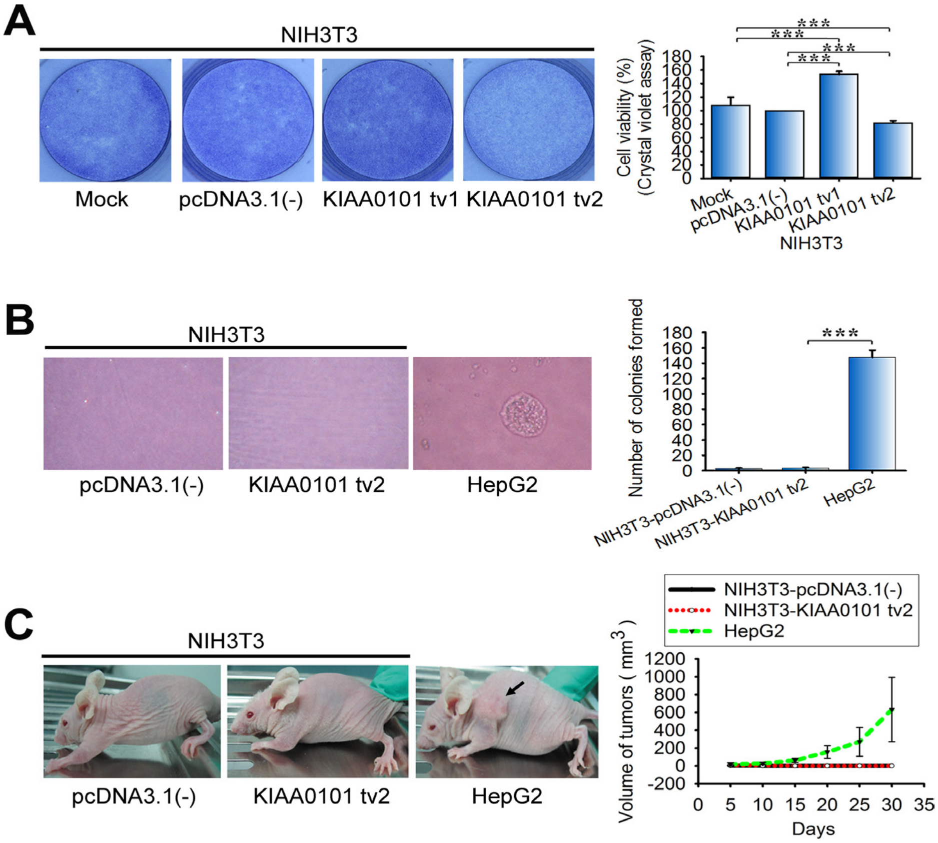

Supplementary Figure 1: KIAA0101 tv2, unlike KIAA0101 tv1, fails to promote NIH3T3 cells proliferation and transformation. Cultured NIH3T3 cells were transfected with KIAA0101 tv1 or tv2 plasmid, the empty vector was used as the negative control. **(A)** Cell viability was determined 48 h after transfection using crystal violet assay. **(B)** Colony formation assay. The graph shows the number of colony in stable KIAA0101 tv2-transfected NIH3T3 cells. HepG2 cells were used as positive control. **(C)** Tumor xenograft assay. The graph shows the mean±SD of tumor volume in stable KIAA0101 tv2-transfected NIH3T3 cells. HepG2 was used as positive control. The arrow indicates the xenograft tumor. All Columns represent the mean±SD of at least three independent experiments. ****P*<0.005.

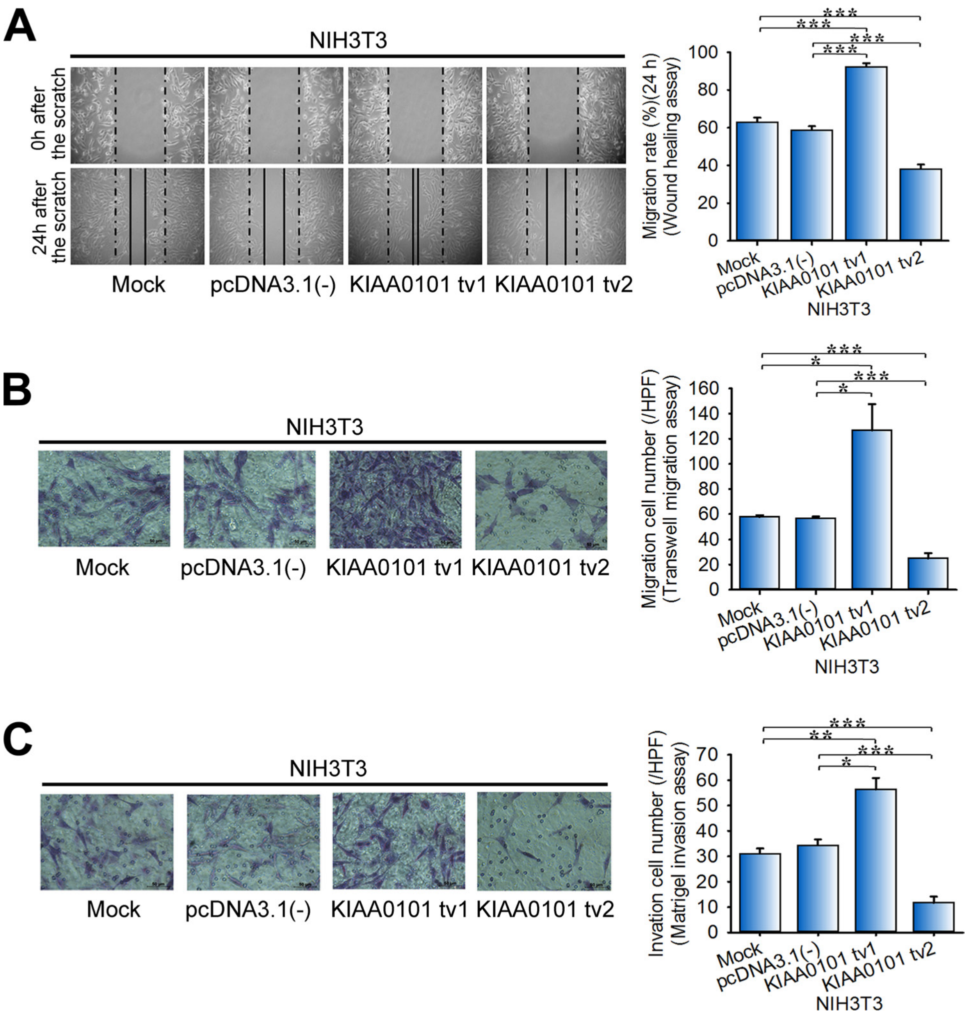

Supplementary Figure 2: Overexpression of KIAA0101 tv2 was contrary to KIAA0101 tv1 in NIH3T3 cells migration and invasion. **(A)** Wound healing assay. The effects of KIAA0101 variants on the wound closure of NIH3T3 cells were determined. Representative wound-closing cells (24 h) after onset of scratching (0 h) are shown. **(B)** Transwell migration assay and **(C)** Matrigel invasion assay were performed using transwell. Representative images of crystal violet-stained cells on the membrane are shown (400×). The bars represent the mean±SD of the migrated or invasion cell numbers. *$P<0.05$, **$P<0.01$, ***$P<0.005$.

Supplementary Table 1: Volume of tumors (mm^3) of nude mice at the 30th day after injection

Groups	Volume of tumors (mm^3) of nude mice at the 30th day				
	1	2	3	4	5
pcDNA3.1(-)	863.0000	863.0000	13.5000	767.9000	960.7560
KIAA0101 tv2	352.0000	171.5000	0.0000	0.0000	46.2300
sh(-)	625.0000	767.9000	13.5000	863.0000	889.0000
shKIAA0101 tv1	0.0000	0.0000	0.0000	26.4000	13.5000

Supplementary Table 2: Sequences of forward and reverse primers used in this study

Primer	5′-3′
P1-Forword (underlined is *BamH* I site)	AGGGATCCAACATGGTGCGGACT
P1-Reverse (underlined is *Hind* III site)	CCCAAGCTTTTATTCAAAGATG
P2-Forword (underlined is *Kpn* I site)	GGGGTACCCCATTCGGTTCTCAAG
P2-Reverse (underlined is *Xho* I site)	CCGCTCGAGGGGTGTTTCACT
P3-Forword	AGAGCTACGAGCTGCCTGAC
P3-Reverse	AGCACTGTGTTGGCGTACAG
P4-Forword	AACTCCCAAGTGGCAAAAAGG
P4-Reverse	CAGGGTAAACAAGGAGACGTT
P5-Forword	CCTCAGCATCTTATCCGAGTGG
P5-Reverse	TGGATGGTGGTACAGTCAGAGC
P6-Forword	TCAGGATGCGTCCACCAAGAAG
P6-Reverse	TGTGTCCACGGCGGCAATCATC
P7-Forword	CTGGAAGTCGAGTGTGCTACTC
P7-Reverse	TGAAGGAGTCCCCTCATGCAAG
P8-Forword	ACGACCTCAACGCACAGTACGA
P8-Reverse	CCTAATTGGGCTCCATCTCGGG
P9-Forword	AGGTGGACCTGGAGACTCTCAG
P9-Reverse	TCCTCTTGGAGAAGATCAGCCG
